# 1,2,4,5-Tetra­fluoro-3,6-diiodo­benzene–4-(pyridin-4-ylsulfan­yl)pyridine (1/1)

**DOI:** 10.1107/S1600536810038316

**Published:** 2010-09-30

**Authors:** Hadi D. Arman, Trupta Kaulgud, Edward R. T. Tiekink

**Affiliations:** a Department of Chemistry, The University of Texas at San Antonio, One UTSA Circle, San Antonio, Texas 78249-0698, USA; b Department of Chemistry, University of Malaya, 50603 Kuala Lumpur, Malaysia

## Abstract

The asymmetric unit of the title 1:1 adduct, C_10_H_8_N_2_S·C_6_F_4_I_2_, comprises a half-mol­ecule of 1,2,4,5-tetra­fluoro-3,6-diiodo­benzene, and half a 4-(pyridin-4-ylsulfan­yl)pyridine mol­ecule. The former is completed by crystallographic inversion symmetry, the latter by twofold symmetry, with the S atom lying on the rotation axis. The almost planar 1,2,4,5-tetra­fluoro-3,6-diiodo­benzene mol­ecule (r.m.s. deviation of all 12 atoms = 0.016 Å) and twisted 4-(pyridin-4-ylsulfan­yl)pyridine mol­ecule [dihedral angle between pyridyl rings = 54.88 (13)°] are connected by N⋯I inter­actions [2.838 (4) Å], generating a supra­molecular chain with a step-ladder topology. These chains are connected in the crystal by C—H⋯F and C—H⋯π(pyrid­yl) inter­actions.

## Related literature

For related studies on co-crystal formation, see: Broker *et al.* (2008 ▶); Arman *et al.* (2010 ▶). For background to halogen bonding, see: Metrangolo *et al.* (2008 ▶); Pennington *et al.* (2008 ▶). For the desulfurization of 4-(pyridin-4-yldisulfan­yl)pyridine, see: Aragoni *et al.* (2007 ▶).
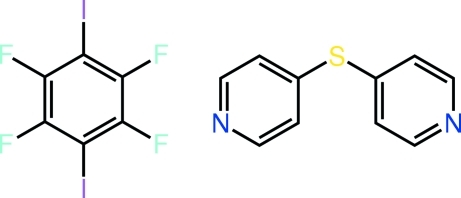


## Experimental

### 

#### Crystal data

C_10_H_8_N_2_S·C_6_F_4_I_2_
*M* *_r_* = 590.10
Monoclinic, 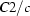

*a* = 13.804 (5) Å
*b* = 5.829 (2) Å
*c* = 22.164 (8) Å
β = 97.989 (7)°
*V* = 1766.1 (11) Å^3^
*Z* = 4
Mo *K*α radiation
μ = 3.72 mm^−1^
*T* = 98 K
0.30 × 0.20 × 0.05 mm

#### Data collection

Rigaku AFC12/SATURN724 diffractometer
Absorption correction: multi-scan (*ABSCOR*; Higashi, 1995 ▶) *T*_min_ = 0.757, *T*_max_ = 1.000
5209 measured reflections
1823 independent reflections
1733 reflections with *I* > 2σ(*I*)
*R*_int_ = 0.027

#### Refinement

*R*[*F* ^2^ > 2σ(*F* ^2^)] = 0.034
*wR*(*F* ^2^) = 0.073
*S* = 1.07
1823 reflections
114 parameters
H-atom parameters constrained
Δρ_max_ = 1.34 e Å^−3^
Δρ_min_ = −0.63 e Å^−3^

### 

Data collection: *CrystalClear* (Molecular Structure Corporation & Rigaku, 2005 ▶); cell refinement: *CrystalClear*; data reduction: *CrystalClear*; program(s) used to solve structure: *SHELXS97* (Sheldrick, 2008 ▶); program(s) used to refine structure: *SHELXL97* (Sheldrick, 2008 ▶); molecular graphics: *ORTEP-3* (Farrugia, 1997 ▶) and *DIAMOND* (Brandenburg, 2006 ▶); software used to prepare material for publication: *publCIF* (Westrip, 2010 ▶).

## Supplementary Material

Crystal structure: contains datablocks global, I. DOI: 10.1107/S1600536810038316/hb5647sup1.cif

Structure factors: contains datablocks I. DOI: 10.1107/S1600536810038316/hb5647Isup2.hkl

Additional supplementary materials:  crystallographic information; 3D view; checkCIF report

## Figures and Tables

**Table 1:** Hydrogen-bond geometry (Å, °) *Cg*1 is the centroid of the N1,C4–C8 ring.

*D*—H⋯*A*	*D*—H	H⋯*A*	*D*⋯*A*	*D*—H⋯*A*
C5—H5⋯F1^i^	0.95	2.52	3.213 (5)	130
C8—H8⋯*Cg*1^ii^	0.95	2.82	3.557 (5)	135

Symmetry codes: (i) 
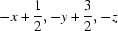
; (ii) 
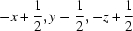
.

**Fig. 1.**
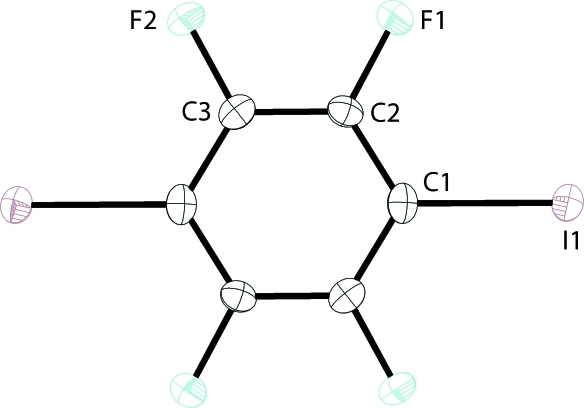
Molecular structure of 1,2,4,5-tetrafluoro-3,6-diiodobenzene found in the structure of (I) showing displacement ellipsoids at the 50% probability level. Unlabelled atoms are related across a centre of inversion.

**Fig. 2.**
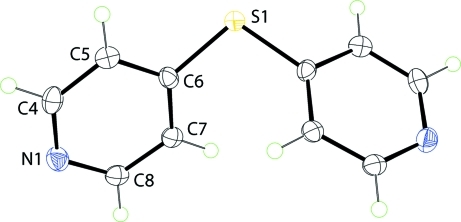
Molecular structure of 4-(pyridin-4-yldisulfanyl)pyridine found in the structure of (I) showing displacement ellipsoids at the 50% probability level. Unlabelled atoms are related across a 2-fold axis.

**Fig. 3.**
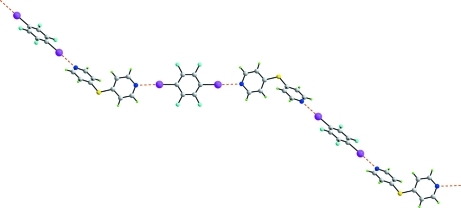
The supramolecular chain in (I) sustained by N···I halogen bonds, shown as orange dashed lines.

**Fig. 4.**
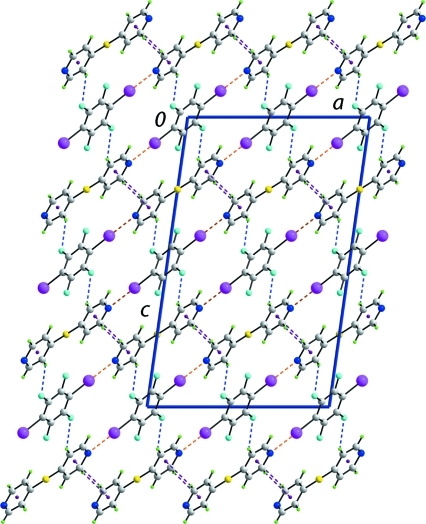
A view in projection down the b axis showing the stacking of alternating layers of 1,2,4,5-tetrafluoro-3,6-diiodobenzene and 4-(pyridin-4-yldisulfanyl)pyridine molecules along the c axis. The N···I, C—H···F and C—H···π interactions are shown as orange, blue and purple dashed lines, respectively.

## supplementary crystallographic information

### Comment

As a continuation of studies into the phenomenon of co-crystallization (Broker *et al.*, 2008; Arman *et al.*, 2010), including investigations of halogen bonding (Pennington *et al.*, 2008), the co-crystallization of 1,2,4,5-tetrafluoro-3,6-diiodobenzene and 4-(pyridin-4-yldisulfanyl)pyridine was investigated. This lead to the isolation of the title 1/1 co-crystal, (I), in which desulfurization of 4-(pyridin-4-yldisulfanyl)pyridine has occurred, a process that has literature precedents (Aragoni *et al.*, 2007).

The asymmetric unit in (I) comprises half a molecule of 1,2,4,5-tetrafluoro-3,6-diiodobenzene as this is situated about a centre of inversion, Fig. 1, and half a molecule of 4-(pyridin-4-ylsulfanyl)pyridine, Fig. 2, with the S atom lying on a 2-fold axis. The C_6_F_4_I_2_ molecule is planar with the r.m.s. deviation of all 12 atoms being 0.016 Å. The pyridyl rings in 4-(pyridin-4-ylsulfanyl)pyridine are twisted and form a dihedral angle of 54.88 (13) °.

The components of the co-crystal are connected *via* N···I interactions [2.838 (4) Å] to form a supramolecular chain with a step-ladder topology and with a base vector 2 0 1, Fig. 3. The chains are consolidated in the crystal packing by C—H···F and C—H···π(pyridyl) interactions, Table 1 and Fig. 4. The N···I interactions observed in (I) represents a further example of N···I—C halogen bonding (Metrangolo *et al.*, 2008).

### Experimental

Initially 1,2,4,5-tetrafluoro-3,6-diiodobenzene (Aldrich, 0.04 mmol) and 4-(pyridin-4-yldisulfanyl)pyridine (Aldrich, 0.04 mmol) were dissolved in a THF/acetone (1/1) mixture and after evaporation of the solvent, the powder was then dissolved in ethanol. Again, crystals did not form so the powder was dissolved in a CHCl_3_/acetone (1/1) mixture. Slow evaporation of this solution deposited yellow blocks of (I) which, after crystallographic characterization, was proven to contain 4-(pyridin-4-ylsulfanyl)pyridine, indicating that desulfurization of 4-(pyridin-4-yldisulfanyl)pyridine had occurred (Aragoni *et al.*, 2007). *M*. pt:. 423–427 K. IR assignment (cm^-1^): 757 (m, sh); 807 (s, sh); 939 (s, sh); 1065 (s, sh); 1208 (m, sh) (C—F); 1408 (m, sh), 1456 (s, sh) C—C (aromatic); 1570 (s, sh) C═N; 2924 (*s*) C—H.

### Refinement

C-bound H-atoms were placed in calculated positions (C–H 0.95 Å) and were included in the refinement in the riding model approximation with *U*_iso_(H) set to 1.2*U*_eq_(C). The maximum and minimum residual electron density peaks of 1.34 and 0.63 e Å^-3^, respectively, were located 1.06 Å and 0.88 Å from the S1 and I1 atoms, respectively.

### Crystal data

**Table:**

C_10_H_8_N_2_S·C_6_F_4_I_2_	*F*(000) = 1104
*M**_r_* = 590.10	*D*_x_ = 2.219 Mg m^−^^3^
Monoclinic, *C*2/*c*	Mo *K*α radiation, λ = 0.71073 Å
Hall symbol: -C 2yc	Cell parameters from 3043 reflections
*a* = 13.804 (5) Å	θ = 3.0–40.2°
*b* = 5.829 (2) Å	µ = 3.72 mm^−^^1^
*c* = 22.164 (8) Å	*T* = 98 K
β = 97.989 (7)°	Block, yellow
*V* = 1766.1 (11) Å^3^	0.30 × 0.20 × 0.05 mm
*Z* = 4	

### Data collection

**Table:**

Rigaku AFC12K/SATURN724 diffractometer	1823 independent reflections
Radiation source: fine-focus sealed tube	1733 reflections with *I* > 2σ(*I*)
graphite	*R*_int_ = 0.027
ω scans	θ_max_ = 26.5°, θ_min_ = 3.0°
Absorption correction: multi-scan (*ABSCOR*; Higashi, 1995)	*h* = −17→14
*T*_min_ = 0.757, *T*_max_ = 1.000	*k* = −5→7
5209 measured reflections	*l* = −27→26

### Refinement

**Table:**

Refinement on *F*^2^	Primary atom site location: structure-invariant direct methods
Least-squares matrix: full	Secondary atom site location: difference Fourier map
*R*[*F*^2^ > 2σ(*F*^2^)] = 0.034	Hydrogen site location: inferred from neighbouring sites
*wR*(*F*^2^) = 0.073	H-atom parameters constrained
*S* = 1.07	*w* = 1/[σ^2^(*F*_o_^2^) + (0.0202*P*)^2^ + 21.2619*P*] where *P* = (*F*_o_^2^ + 2*F*_c_^2^)/3
1823 reflections	(Δ/σ)_max_ = 0.001
114 parameters	Δρ_max_ = 1.34 e Å^−^^3^
0 restraints	Δρ_min_ = −0.63 e Å^−^^3^

### Special details

**Table:**

Geometry. All s.u.'s (except the s.u. in the dihedral angle between two l.s. planes) are estimated using the full covariance matrix. The cell s.u.'s are taken into account individually in the estimation of s.u.'s in distances, angles and torsion angles; correlations between s.u.'s in cell parameters are only used when they are defined by crystal symmetry. An approximate (isotropic) treatment of cell s.u.'s is used for estimating s.u.'s involving l.s. planes.
Refinement. Refinement of *F*^2^ against ALL reflections. The weighted *R*-factor *wR* and goodness of fit *S* are based on *F*^2^, conventional *R*-factors *R* are based on *F*, with *F* set to zero for negative *F*^2^. The threshold expression of *F*^2^ > 2σ(*F*^2^) is used only for calculating *R*-factors(gt) *etc*. and is not relevant to the choice of reflections for refinement. *R*-factors based on *F*^2^ are statistically about twice as large as those based on *F*, and *R*- factors based on ALL data will be even larger.

### Fractional atomic coordinates and isotropic or equivalent isotropic displacement parameters (Å^2^)

**Table:**

	*x*	*y*	*z*	*U*_iso_*/*U*_eq_	
I1	0.33988 (2)	0.75767 (5)	0.089898 (12)	0.02060 (11)	
F1	0.4107 (2)	0.9003 (5)	−0.03751 (12)	0.0253 (6)	
F2	0.5292 (2)	0.6993 (5)	−0.10663 (12)	0.0257 (6)	
C1	0.4356 (3)	0.6036 (8)	0.0360 (2)	0.0186 (9)	
C2	0.4540 (3)	0.6999 (7)	−0.0181 (2)	0.0180 (9)	
C3	0.5168 (3)	0.5980 (8)	−0.0534 (2)	0.0205 (9)	
S1	0.0000	0.4266 (3)	0.2500	0.0201 (3)	
N1	0.2103 (3)	−0.0202 (7)	0.15987 (18)	0.0248 (9)	
C4	0.1787 (3)	0.1842 (9)	0.1379 (2)	0.0239 (10)	
H4	0.2032	0.2404	0.1028	0.029*	
C5	0.1121 (3)	0.3192 (8)	0.1635 (2)	0.0215 (9)	
H5	0.0905	0.4616	0.1455	0.026*	
C6	0.0779 (3)	0.2417 (8)	0.2158 (2)	0.0191 (9)	
C7	0.1112 (3)	0.0326 (8)	0.2401 (2)	0.0193 (9)	
H7	0.0900	−0.0242	0.2763	0.023*	
C8	0.1762 (3)	−0.0926 (8)	0.2104 (2)	0.0203 (9)	
H8	0.1977	−0.2373	0.2268	0.024*	

### Atomic displacement parameters (Å^2^)

**Table:**

	*U*^11^	*U*^22^	*U*^33^	*U*^12^	*U*^13^	*U*^23^
I1	0.01922 (17)	0.02358 (17)	0.01968 (17)	0.00034 (12)	0.00505 (11)	−0.00148 (11)
F1	0.0271 (15)	0.0212 (14)	0.0285 (15)	0.0061 (12)	0.0072 (12)	0.0045 (11)
F2	0.0288 (16)	0.0283 (14)	0.0218 (14)	0.0026 (12)	0.0099 (11)	0.0084 (11)
C1	0.013 (2)	0.023 (2)	0.020 (2)	−0.0024 (17)	0.0021 (16)	−0.0062 (17)
C2	0.015 (2)	0.017 (2)	0.021 (2)	−0.0005 (17)	−0.0009 (16)	0.0016 (16)
C3	0.021 (2)	0.023 (2)	0.018 (2)	−0.0036 (19)	0.0029 (17)	0.0006 (17)
S1	0.0203 (8)	0.0166 (7)	0.0245 (8)	0.000	0.0068 (6)	0.000
N1	0.020 (2)	0.026 (2)	0.028 (2)	0.0012 (17)	0.0057 (16)	−0.0018 (17)
C4	0.017 (2)	0.031 (3)	0.023 (2)	−0.0047 (19)	0.0040 (18)	0.0002 (19)
C5	0.022 (2)	0.020 (2)	0.022 (2)	−0.0007 (18)	0.0005 (18)	0.0007 (17)
C6	0.015 (2)	0.020 (2)	0.023 (2)	−0.0012 (17)	0.0041 (17)	−0.0043 (17)
C7	0.014 (2)	0.019 (2)	0.024 (2)	−0.0025 (17)	0.0003 (17)	−0.0017 (17)
C8	0.016 (2)	0.018 (2)	0.026 (2)	−0.0010 (17)	−0.0013 (17)	−0.0009 (17)

### Geometric parameters (Å, °)

**Table:**

I1—C1	2.101 (4)	N1—C4	1.337 (6)
F1—C2	1.355 (5)	C4—C5	1.389 (7)
F2—C3	1.352 (5)	C4—H4	0.9500
C1—C2	1.379 (6)	C5—C6	1.387 (6)
C1—C3^i^	1.375 (6)	C5—H5	0.9500
C2—C3	1.381 (6)	C6—C7	1.385 (6)
C3—C1^i^	1.375 (6)	C7—C8	1.392 (6)
S1—C6^ii^	1.766 (4)	C7—H7	0.9500
S1—C6	1.766 (4)	C8—H8	0.9500
N1—C8	1.342 (6)		
			
C2—C1—C3^i^	116.9 (4)	C5—C4—H4	118.0
C2—C1—I1	121.8 (3)	C6—C5—C4	118.5 (4)
C3^i^—C1—I1	121.3 (3)	C6—C5—H5	120.7
C1—C2—F1	120.0 (4)	C4—C5—H5	120.7
C1—C2—C3	121.6 (4)	C7—C6—C5	118.6 (4)
F1—C2—C3	118.4 (4)	C7—C6—S1	124.0 (4)
F2—C3—C1^i^	120.3 (4)	C5—C6—S1	117.3 (3)
F2—C3—C2	118.2 (4)	C6—C7—C8	118.6 (4)
C1^i^—C3—C2	121.5 (4)	C6—C7—H7	120.7
C6^ii^—S1—C6	104.8 (3)	C8—C7—H7	120.7
C8—N1—C4	116.7 (4)	N1—C8—C7	123.7 (4)
N1—C4—C5	123.9 (4)	N1—C8—H8	118.2
N1—C4—H4	118.0	C7—C8—H8	118.2
			
C3^i^—C1—C2—F1	−179.1 (4)	N1—C4—C5—C6	1.7 (7)
I1—C1—C2—F1	0.6 (6)	C4—C5—C6—C7	−0.3 (7)
C3^i^—C1—C2—C3	0.4 (7)	C4—C5—C6—S1	175.7 (4)
I1—C1—C2—C3	−179.9 (3)	C6^ii^—S1—C6—C7	−34.9 (3)
C1—C2—C3—F2	178.2 (4)	C6^ii^—S1—C6—C5	149.3 (4)
F1—C2—C3—F2	−2.3 (6)	C5—C6—C7—C8	−1.1 (6)
C1—C2—C3—C1^i^	−0.4 (8)	S1—C6—C7—C8	−176.8 (3)
F1—C2—C3—C1^i^	179.0 (4)	C4—N1—C8—C7	0.0 (7)
C8—N1—C4—C5	−1.5 (7)	C6—C7—C8—N1	1.3 (7)

Symmetry codes: (i) −*x*+1, −*y*+1, −*z*; (ii) −*x*, *y*, −*z*+1/2.

### Hydrogen-bond geometry (Å, °)

**Table:**

Cg1 is the centroid of the N1,C4–C8 ring.

**Table:**

*D*—H···*A*	*D*—H	H···*A*	*D*···*A*	*D*—H···*A*
C5—H5···F1^iii^	0.95	2.52	3.213 (5)	130
C8—H8···Cg1^iv^	0.95	2.82	3.557 (5)	135

Symmetry codes: (iii) −*x*+1/2, −*y*+3/2, −*z*; (iv) −*x*+1/2, *y*−1/2, −*z*+1/2.

## References

[bb1] Aragoni, M. C., Arca, M., Crespo, M., Devillanova, F. A., Hursthouse, M. B., Huth, S. L., Isaia, F., Lippolis, V. & Verani, G. (2007). *CrystEngComm*, **9**, 873–878.

[bb2] Arman, H. D., Kaulgud, T. & Tiekink, E. R. T. (2010). *Acta Cryst.* E**66**, o2356. 10.1107/S1600536810032721 PMC3008031 21588698

[bb3] Brandenburg, K. (2006). *DIAMOND* Crystal Impact GbR, Bonn, Germany.

[bb4] Broker, G. A., Bettens, R. P. A. & Tiekink, E. R. T. (2008). *CrystEngComm*, **10**, 879–887.

[bb5] Farrugia, L. J. (1997). *J. Appl. Cryst.* **30**, 565.

[bb6] Higashi, T. (1995). *ABSCOR* Rigaku Corporation, Tokyo, Japan.

[bb7] Metrangolo, P., Resnati, G., Pilati, T. & Biella, S. (2008). *Struct. Bond.* **126**, 105–136.

[bb8] Molecular Structure Corporation & Rigaku (2005). *CrystalClear* MSC, The Woodlands, Texas, USA, and Rigaku Corporation, Tokyo, Japan.

[bb9] Pennington, W. T., Hanks, T. W. & Arman, H. D. (2008). *Struct. Bond.* **126**, 65–104.

[bb10] Sheldrick, G. M. (2008). *Acta Cryst.* A**64**, 112–122. 10.1107/S0108767307043930 18156677

[bb11] Westrip, S. P. (2010). *J. Appl. Cryst.* **43**, 920–925.

